# A Two-Stream Deep Fusion Framework for High-Resolution Aerial Scene Classification

**DOI:** 10.1155/2018/8639367

**Published:** 2018-01-18

**Authors:** Yunlong Yu, Fuxian Liu

**Affiliations:** Air Defense and Anti-Missile College, Air Force Engineering University, Xi'an 710051, China

## Abstract

One of the challenging problems in understanding high-resolution remote sensing images is aerial scene classification. A well-designed feature representation method and classifier can improve classification accuracy. In this paper, we construct a new two-stream deep architecture for aerial scene classification. First, we use two pretrained convolutional neural networks (CNNs) as feature extractor to learn deep features from the original aerial image and the processed aerial image through saliency detection, respectively. Second, two feature fusion strategies are adopted to fuse the two different types of deep convolutional features extracted by the original RGB stream and the saliency stream. Finally, we use the extreme learning machine (ELM) classifier for final classification with the fused features. The effectiveness of the proposed architecture is tested on four challenging datasets: UC-Merced dataset with 21 scene categories, WHU-RS dataset with 19 scene categories, AID dataset with 30 scene categories, and NWPU-RESISC45 dataset with 45 challenging scene categories. The experimental results demonstrate that our architecture gets a significant classification accuracy improvement over all state-of-the-art references.

## 1. Introduction

Aerial scene classification is a key problem in aerial image understanding, which aims to automatically assign a semantic label to each aerial image in order to know which category it belongs to [1, 2]. Aerial scene classification has important application value in military and civil areas such as disaster monitoring, weapon guidance, and traffic supervision [3, 4]. Aerial images not only have rich space and texture features but also contain a large number of scene semantic information. However, since the composition of the scene is complicated, it is difficult to obtain the scene information of interest directly from the massive image data [5, 6].

In order to understand and identify the scene information in aerial images, many scene classification methods are proposed; they generally can be divided into two categories: methods with low-level scene features and methods with midlevel scene features. The commonly used low-level methods include Scale Invariant Feature Transform (SIFT) [7], Local Binary Pattern (LBP) [8], Color Histogram (CH) [9], and GIST [10]. The midlevel methods represent a scene by coding the low-level local feature descriptors. The midlevel coding methods include Bag of Visual Words (BoVW) [11], Spatial Pyramid Matching (SPM) [12], Locality-Constrained Linear Coding (LLC) [13], Probabilistic Latent Semantic Analysis (PLSA) [14], Latent Dirichlet Allocation (LDA) [15], Improved Fisher Kernel (IFK) [16], and Vector of Locally Aggregated Descriptors (VLAD) [17].

In recent years, the deep learning methods have a breakthrough in computer vision tasks, such as image classification, object recognition, and face recognition [18–20]. Convolutional neural network (CNN) is one of the most successful deep learning algorithms. Recently, CNN models, such as CaffeNet [21] and GoogLeNet [22], achieve better performance on aerial scene classification than that of low-level and midlevel methods.

A typical architecture of CNN usually contains many layers to automatically extract useful features and exploit the logistic regression for classification. However, this classifier cannot reach a satisfactory prediction performance. To solve this problem, CNN-SVM [23] was proposed. This architecture is a combination of CNN and support vector machine (SVM), which uses pretrained CNN as feature extractor and SVM as a classifier. Inspired by its success, some new combination architectures were proposed, such as CNN-BPR [24].

Extreme learning machine (ELM) is a learning algorithm based on single-hidden layer feedforward neural network (SLFN) [25]. According to its creators, this model is able to produce good generalization performance and learn thousands of times faster than networks trained using backpropagation. In [26], it also shows that the ELM can outperform SVM. In [27], the authors have confirmed that the CNN-ELM outperforms CNN-SVM in the area of high-resolution aerial scene classification. Therefore, ELM with CNN-learned features can perform excellently.

In this paper, we propose a new aerial scene classification framework that combines the fused deep convolutional features learned by CNNs with the ELM classifier. First, two pretrained CNNs are used as feature extractor to learn deep features from the original aerial image and the processed aerial image through saliency detection, respectively. Second, these two sets of features extracted by the original RGB stream and the saliency stream are fused to one set of features. Finally, the ELM classifier is used for final classification with the fused features. Experimental results on four datasets illustrate that the proposed architecture outperforms the sate-of-the-art methods.

The contributions of this paper are concluded as follows.

(1) We employ a two-stream deep architecture to extract features from the original aerial image and the processed aerial image through saliency detection, respectively. Thus, we can get two different types of deep convolutional features which contain the appearance information and prominent information.

(2) To the best of our knowledge, it is the first to fuse these two different types of deep convolutional features extracted by the original RGB stream and the saliency stream, which can get a good representation of the aerial images.

(3) We use the extreme learning machine as a classifier for final classification with the fused features.

The rest of this paper is organized as follows.  Section 2 introduces the related works including convolutional neural networks and extreme learning machine.  Section 3 describes the proposed two-stream deep fusion architecture in detail.  Section 4 evaluates the performance of the proposed architecture on four different benchmark datasets and makes comparisons with several state-of-the-art methods. The conclusions are drawn in Section 5.

## 2. Related Works

### 2.1. Convolutional Neural Networks

As a branch of machine learning, deep learning is a calculation model consisting of multiple processing layers. Much attention has been paid to deep learning for its great breakthrough in fields including image classification, voice understanding, and video analysis.

Deep convolutional neural network is an important algorithm in field of deep learning. It is based on the classical convolution neural network devised by LeCun [28].

**Figure 1:**
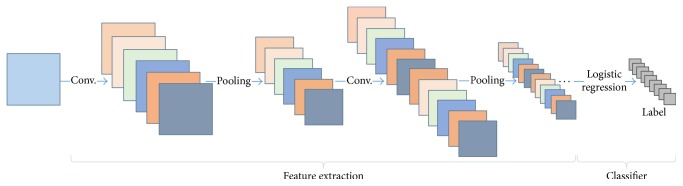
The architecture of DCNN.

In general, DCNN (deep convolutional neural network) consists of two major parts (see Figure 1). The first part is feature extraction, which contains alternating convolutional and pooling layers. A convolutional layer consists of two sublayers: convolutional filter layer and feature mapping layer. Descriptions of the layers are given as follows.

(*1) Convolutional Filter Layer.* Convolution is a kind of linear operation. Noise reduction and characteristic enhancement can be achieved by using the layer for extraction of characteristics. Local characteristics can be extracted by the connection between the input of each neuron and local receptive field of the previous layer. Assume the input image *I* is a two-dimensional image with size of *r* × *r*; an output with size of ((*r* − *w*)/*s* + 1)×((*r* − *w*)/*s* + 1) can be obtained by the convolutional operation of a trainable filter set *K* with size of *w* × *w*:

(1) $$\begin{array}{c} y_{i}=b_{i}+\sum_{i}k_{ij}*x_{i}, \end{array}$$

where *∗* denotes convolutional operation, *x*_*i*_ denotes the input of convolutional layer, *k*_*ij*_ is the parameter of convolutional kernel, *b*_*i*_ is the bias, and *s* represents step length; each filter is related to a certain feature.

(*2) Feature Mapping Layer.* A nonlinear activation function is used for mapping of results obtained from filter layer, thus generating feature graph *F*.

(2) $$\begin{array}{c} f_{s}=\sigma \left(\;b_{i}+\sum_{i}k_{ij}*x_{i}\;\right), \end{array}$$

where *σ* is a nonlinear activation function. Traditional activation functions include tanh, sigmoid, and softplus. ReLU (Restricted Linear Units) is the closest one to the activation model of stimulated biological neuron, thus gradually being used as activation function of neural networks.

(*3) Pooling Layer.* This layer is used for elimination of redundant data. After dividing the feature graph *F* into *m* × *m* nonintersectional areas, pooling features *P* with size of {((*r* − *w*)/*s* + 1)/*m*} × {((*r* − *w*)/*s* + 1)/*m*} can be obtained based on statistical mean value (or maximum value) of the separate regions. Dimensions of the feature can be greatly reduced after the pooling procedure, thus avoiding overfitting and enabling the models to be robust.

Acting as a combined effort to extract features of the input image, convolutional filter layer, feature mapping layer, and pooling layer are considered as one layer in the DCNN. After several layers of convolution and pooling, the input image is represented by some learned features.

The second part is classifier. The learned features can be put into the logistic regression classifier for classification. The logistic regression classifier uses softmax as its output-layer activation function.

The network parameters are trained by BP (backpropagation) algorithm [29] with SGD (Stochastic Gradient Descent). Dropout strategy [30] is applied to avoid overfitting and enhance the generalization ability of the networks. The dropout strategy is usually used in fully connected layers.

### 2.2. Extreme Learning Machine

**Figure 2:**
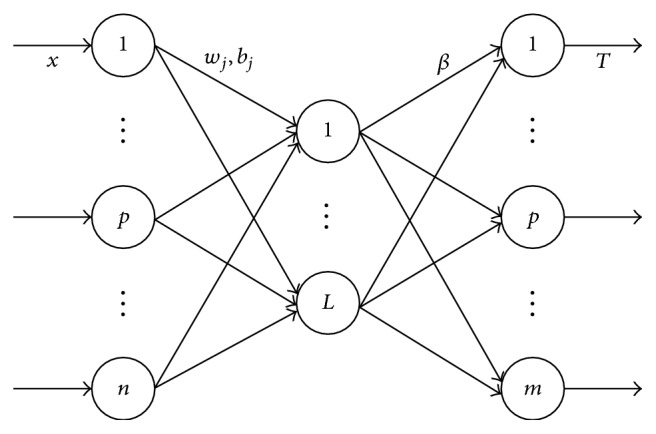
The structure of the ELM.

Extreme learning machine consists of three layers: input layer, hidden layer, and output layer. The structure of the ELM is shown in Figure 2.

With regard to *N* different samples (*x*_*i*_, *t*_*i*_), *x*_*i*_ = [*x*_*i*1_, *x*_*i*2_,…, *x*_*in*_]^*T*^ denotes the *i*th sample and *t*_*i*_ = [*t*_*i*1_, *t*_*i*2_,…, *t*_*im*_]^*T*^ denotes the actual label of the *i*th sample. The number of input nodes *n* is the dimension of each sample; the number of output nodes *m* is total number of categories. Given *L* hidden nodes and activation function *g*(*x*), there must exist a set of parameters *w*_*j*_, *b*_*j*_, and *β*_*j*_, which can make this network approach these *N* different samples.

(3) $$\begin{array}{c} \overset{L}{\underset{j=1}{\sum }}\beta_{j}g\left(\;w_{j}x_{i}+b_{j}\;\right)=t_{i},\;i=1,2,\ldots ,N, \end{array}$$

where *w*_*j*_ = [*w*_*j*1_, *w*_*j*2_,…, *w*_*jn*_]^*T*^ is the weight vector that connects the *j*th hidden node with the input nodes, *β*_*j*_ = [*β*_*j*1_, *β*_*j*2_,…, *β*_*jm*_]^*T*^ is the weight vector that connects the *j*th hidden node with the output nodes, and *b*_*j*_ is the bias of the *j*th hidden node.

Equation (3) can be simplified as matrix form,

(4) $$\begin{array}{c} H\beta =T, \end{array}$$

where *H* is the output matrix of the hidden layer and the *j*th row of *H* is the output of the *j*th hidden node with respect to the input samples *x*_1_, *x*_2_,…, *x*_*N*_.

(5) Hw1,…,wL,b1,…,bL,x1,…,xN=gw1x1+b1⋯gwLx1+bL⋮⋮gw1xN+b1⋯gwLxN+bLβ=β1T⋮βLTL×mT=t1T⋮tNTN×m.

In ELM algorithm, the input weights and the hidden layer biases of SLFN need not be adjusted at all and can be arbitrarily given. With regard to the fixed input weights and the hidden layer biases, we just need to find a least-squares solution $\hat{\beta }$ of the linear system *Hβ* = *T*:

(6) Hw1,…,wL,b1,…,bLβ^−T=minβ⁡Hw1,…,wL,b1,…,bLβ−T.

The minimum norm least-squares solution of the linear system *Hβ* = *T* is

(7) $$\begin{array}{c} \hat{\beta }=H^{†}T, \end{array}$$

where *H*^†^ is the Moore-Penrose generalized inverse of matrix *H*.

## 3. Proposed Architecture

**Figure 3:**
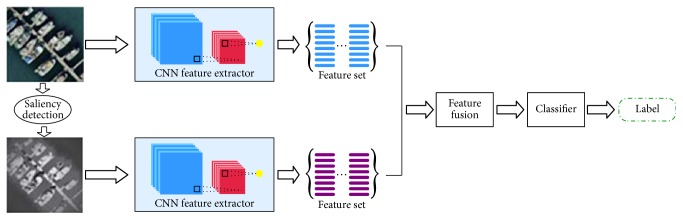
The proposed two-stream deep fusion architecture.

In this section, we propose an effective and efficient two-stream deep fusion architecture for aerial scene classification. The first stream is called original RGB stream, which can capture the appearance information by using original RGB images as input to the network. The second stream is called saliency stream, which can capture the prominent information by using the processed images through saliency detection as input to the network. This two-stream framework uses two same deep convolutional neural networks as feature extractor to describe the original aerial image and the processed aerial image through saliency detection, respectively. Then, we use two famous strategies to fuse the extracted two sets of features. Finally, the fused features are fed into the ELM classifier for aerial scene classification. The overall framework of our proposed method is shown in Figure 3. As described in Figure 3, our proposed architecture includes the following four parts.

(1) Preprocessing the aerial images based on unsupervised saliency detection.

(2) Using the original RGB stream and the saliency stream to extract features from the two kinds of aerial image. These two streams use deep convolutional neural networks to extract features.

(3) Fusing the extracted two sets of features.

(4) Using the ELM classifier for aerial scene classification.

### 3.1. Saliency Detection

When facing visual scenes, human visual system is capable of quickly focusing our eyes on some distinctive visual regions and ignoring plain ones. The selective visual attention mechanism can help human beings observe, think, and make decision quickly and efficiently. The saliency detection model [47] emulated human visual attention can make our architecture more intelligent. By use of saliency detection, we can get more informative features which could dominate the category of the image. However, saliency detection is not suitable for all aerial images. Thus, we adopt the fusion model, which can make good use of each strength.

This method includes two sections. One section is the global perspective which can get a global distribution of visual properties. In this section, a visual vocabulary for the aerial scene is built. Each visual word serves as a single element in depicting the aerial scene. The representation form is the histogram of visual word occurrence.

(8) I=frqWkf,Wkf∈ΩΩ=Wkf=W1color,…,WNcolorcolor;W1texture,…,WNtexturetexture,

where *f* ∈ *F*, *F* = {color, texture}. frq(*W*_*k*_^*f*^) indicates the frequency of occurrence of the visual word *W*_*k*_^*f*^. Then, a weighted factor *φ*_*k*_^*f*^ for each visual word is introduced according to the “repetition suppression principle.”

(9) $$\begin{array}{c} \varphi_{k}^{f}=\frac{1}{frq\left(W_{k}^{f}\right)}. \end{array}$$

The other section in this method is the local perspective. The representation for patch *I*_*m*_ (*I*_*m*_ ∈ *I*) is obtained using the histogram of visual word occurrence. Finally, the saliency value of patch *I*_*m*_ is computed by

(10) $$\begin{array}{c} sal\left(\;I_{m}\;\right)=\underset{f\in F}{\sum }\;\overset{N^{f}}{\underset{k=1}{\sum }}frq^{m}\left(\;W_{k}^{f}\;\right)\cdot \varphi_{k}^{f}, \end{array}$$

where frq^*m*^(*W*_*k*_^*f*^) indicates the frequency of occurrence of the visual word *W*_*k*_^*f*^ for patch *I*_*m*_. *N*_*f*_ denotes the number of color and texture feature words.

### 3.2. Feature Extraction

**Figure 4:**
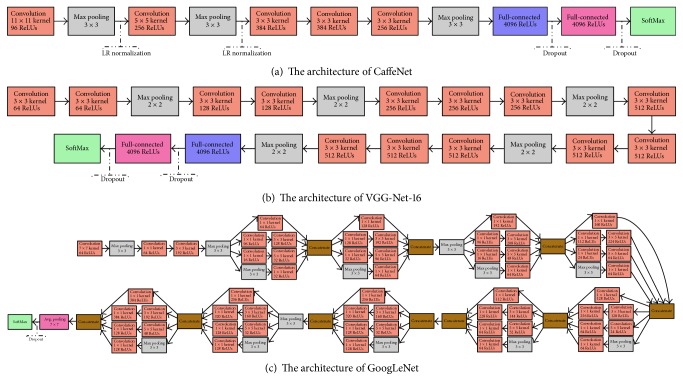
The architectures of different CNNs used in our work.

In recent years, CNN models can get higher classification accuracy than that of low-level and midlevel methods on aerial scene classification. The impressive results of CNNs indicate that the features extracted by CNNs are more typical and representative. Therefore, we select some of the most popular CNN models as feature extractor in our original RGB stream and saliency stream. Three selected CNN architectures are presented in Figure 4. We describe the characteristics of each model in the following part. At the same time, we specify the source of the features for one specific model.

#### 3.2.1. CaffeNet

Caffe (Convolutional Architecture for Fast Feature Embedding) [21] is one of the most popular libraries for deep learning, which is developed by the Berkeley Vision and Learning Center. The network, whose architecture can be seen in Figure 4(a), is almost a replication of AlexNet [48]. However, its training process has no data argumentation and its order of normalization and pooling layers is switched. The architecture of CaffeNet includes five convolutional layers, some of which are followed by max-pooling layers, and three fully connected layers with a softmax. In our architecture, we use CaffeNet as a feature extractor by extracting features from the second fully connected layer, which can get features of 4096 dimensions.

#### 3.2.2. VGG-Net-16

VGG-Net [49] achieves the state-of-the-art accuracy on ILSVRC classification and localization tasks. Due to the use of very small (3 × 3) convolution filters in all layers, the depth of the network can be increased easily by adding more convolutional layers. The authors give five configurations of VGG-Net, whose depth of weight layers is from 16 to 19. In our work, we use the VGG-Net-16 model, whose architecture can be seen in Figure 4(b). This network includes thirteen convolutional layers, five pooling layers, and three fully connected layers with a softmax. In our architecture, we use VGG-Net-16 as a feature extractor by extracting features from the second fully connected layer, which can get features of 4096 dimensions.

#### 3.2.3. GoogLeNet

GoogLeNet [22], proposed by Szegedy et al., is the 22-layer CNN architecture that won the ILSVRC14 competition. The architecture of this network can be seen in Figure 4(c). Its main characteristic is the use of the inception modules, which is derived from the idea of “network in network.” The utilization of the inception modules can make GoogLeNet have two main advantages: (1) in the inception module, the size of filters at the same layer is different, which can get more accurate multiscale spatial information; moreover (2) the design of this module can reduce the number of parameters of the network, which makes the network less prone to overfitting and allows it to be deeper. In fact, the 22-layer GoogLeNet with more than 50 convolutional layers distributed inside the inception modules has approximately five millions of parameters, which is 12 times fewer than that of CaffeNet. In our architecture, we use GoogLeNet as a feature extractor by extracting features from the last pooling layer, which can get features of 1024 dimensions.

### 3.3. Features Fusion

For the original aerial image and the processed aerial image through saliency detection, we use the CNN model pretrained on ImageNet to extract features from the specified layers in the original RGB stream and the saliency stream. The fused features which contain rich information of the image scene can contribute to the process of classification. How to fuse the two different sets of features is becoming an important issue.

Some methods have been proposed for feature fusion [50–52]. We select two classical methods for fusing the two different types of features, in aim to get more informative and significant features to represent the input image.

(1) Serial feature fusion strategy is just to concatenate the two sets of features. The dimension of the fused features is equal to the summation of the dimensions of the two sets of features.

(2) Parallel feature fusion strategy is to combine the two sets of features. Each input image *I* generated two sets of features, that is, *F*_1_ and *F*_2_ representing the two sets of features. The final fused feature representation is formulated as

(11) $$\begin{array}{c} F_{f}\left(\;I\;\right)=F_{1}\left(\;I\;\right)+iF_{2}\left(\;I\;\right), \end{array}$$

where *i* is the imaginary unit.

## 4. Experiments and Analysis

We use the NVIDIA Titan X Pascal GPU (with a 12 GB memory) and 2.0 GHz Intel Xeon CPU E5-2683v3 in this experiment. The proposed architecture is tested on four different datasets. Firstly, we give the description of the four datasets. Secondly, the setup in our experiments is given. Finally, the classification performance of the proposed architecture is compared with the state-of-the-art in the literature.

### 4.1. Datasets

**Figure 5:**
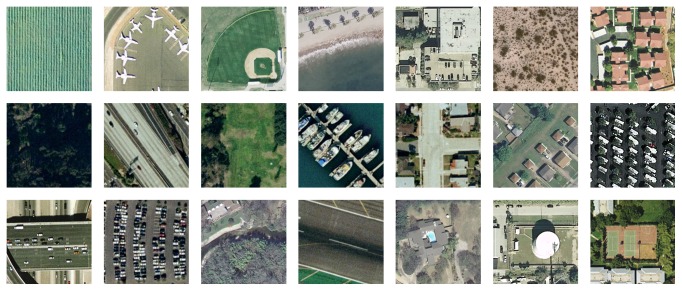
Class representatives of the UC-Merced dataset.

The first dataset is the well-known UC-Merced Land Use dataset [31], which consists of 2100 high-resolution remote sensing images of 21 classes. The size of each image scene is 256 × 256 pixels. The class samples are shown in Figure 5. There are some highly overlapped classes, such as “dense residential,” “medium residential,” and “sparse residential,” which make this dataset difficult for classification. This dataset has been widely used to evaluate different aerial scene classification methods. For more information, visit http://vision.ucmerced.edu/datasets.

**Figure 6:**
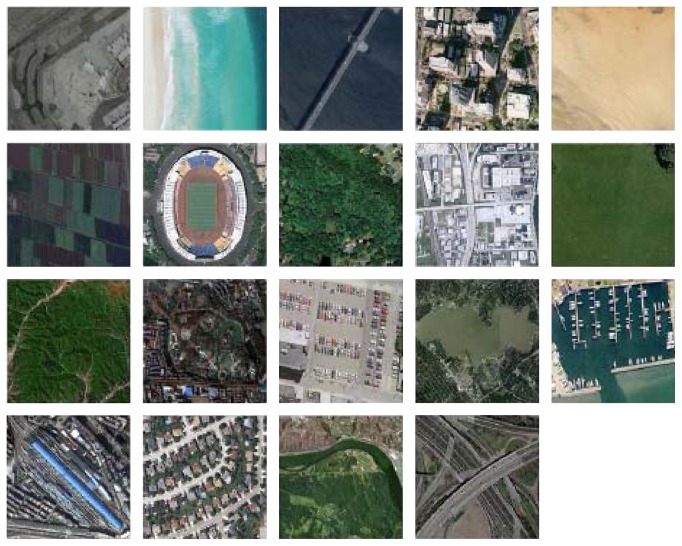
Class representatives of the WHU-RS dataset.

The second dataset is WHU-RS dataset [53], which is collected from Google Earth imagery. There are 950 high-spatial resolution images with 600 × 600 pixels divided into 19 classes. The class samples are shown in Figure 6. The images in this dataset are collected from different regions all over the world, which creates more challenges because of its high diversity. This dataset has also been widely used to evaluate different aerial scene classification methods. For more information, visit http://dsp.whu.edu.cn/cn/staff/yw/HRSscene.html.

**Figure 7:**
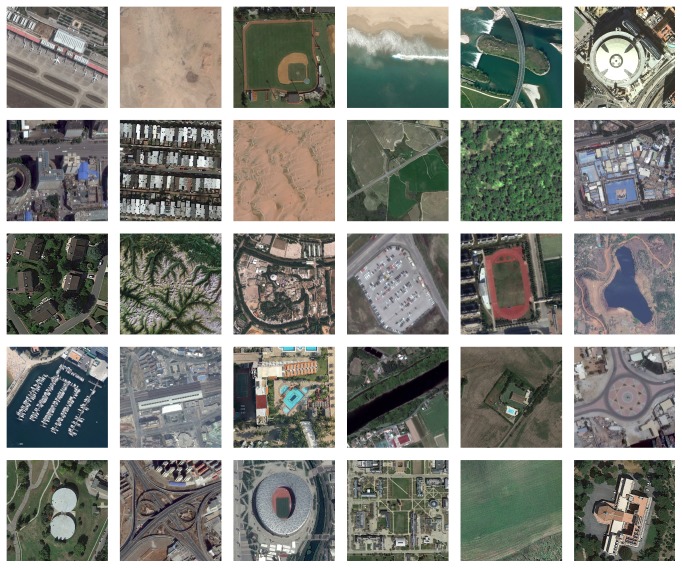
Class representatives of the AID dataset.

The third dataset named AID (a new large-scale aerial image dataset), which is collected from Google Earth imagery [41]. There are a number of 10000 (600 × 600) pixel images within 30 classes in the AID dataset. Compared with other remote sensing image datasets, the AID dataset has some properties which include high intraclass variations, small interclass dissimilarity, and relative large-scale.  Figure 7 shows a sample image for each class included in this dataset. For more information, visit http://www.lmars.whu.edu.cn/xia/AID-project.html.

**Figure 8:**
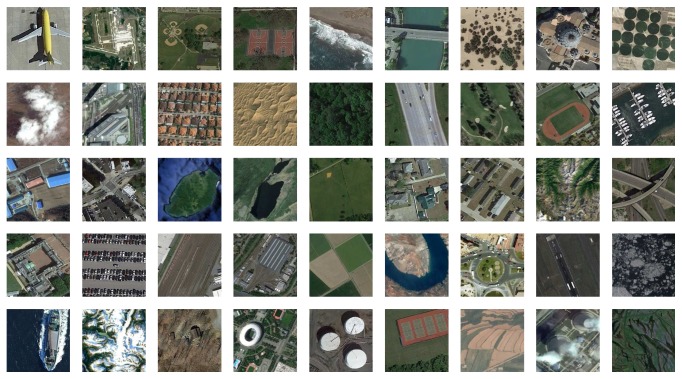
Class representatives of the NWPU-RESISC45 dataset.

The fourth dataset is NWPU-RESISC45 dataset, which contains 31500 images and covers 45 scene classes with 700 images in each class [46].  Figure 8 shows a sample image for each class included in this dataset. For more information, visit http://www.escience.cn/people/JunweiHan/NWPU-RESISC45.html. The AID dataset and the NWPU-RESISC45 dataset are more challenging datasets, which have been used for testing some high performance aerial scene classification methods.

### 4.2. Experimental Setup

For feature extractor selection, we use CaffeNet, VGG-Net-16, and GoogLeNet as feature extractor, respectively. These three networks are all pretrained on ImageNet [54]. After that, we use two fusion strategies to combine among the extracted features. In classification section, we use the extreme learning machine.

With regard to training set generation, we adopt two different settings. For the UC-Merced dataset, the ratio of the number of training set is set to be 50% and 80%, respectively, and the left for testing. For the WHU-RS dataset, the ratios are set to be 40% and 60%, respectively. For the AID dataset, the ratios are set to be 20% and 50%, respectively. For the NWPU-RESISC45 dataset, the ratios are fixed at 10% and 20%, respectively. Considering that CNN requires a predefined size for the input image, all images are resized according to the size of the receptive field of the selected CNN model.

In this paper, we use the overall accuracy to evaluate the methods. The evaluation procedure is repeated ten times for a reliable performance comparison. The final results are reported as the mean and standard deviation over the ten runs. In this section, we do not make comparisons with the results of some fine-tuned networks because our architectures only use the pretrained networks, which is for the sake of fair comparison.

### 4.3. UC-Merced Dataset

With regard to the UC-Merced dataset, we first analyze the influence of different features extractors and fusion strategies on the classification performance. The experimental results are shown in Table 1. In Table 1, we can see that the two-stream architectures provide superior performance compared to the single CNNs without fusion, which illustrates that data fusion is helping the system to increase its accuracy. The serial feature fusion strategy based architectures provide inferior performance compared to the parallel feature fusion strategy based architectures with the same CNN feature extractor. At the same time, we also see that the features extracted by VGG-Net-16 are more representative and discriminative. In this dataset, our best classification accuracy rates are 96.97% and 98.02%, using 50% and 80% training ratios, respectively. These best results are achieved by the architecture that uses VGG-Net-16 network and parallel feature fusion strategy.

We also make a comparison of the proposed architecture against several state-of-the-art aerial scene classification methods on this dataset, as shown in Table 2. As we can see from Table 2, our architecture outperforms all other aerial scene classification methods, with an increase in overall accuracy of 1.08% and 0.60% over the second best model using 50% and 80% training ratios, respectively. The good performance of our method mainly benefits from the fusion of two different types of deep convolutional features and the extreme learning machine.

### 4.4. WHU-RS Dataset

On the WHU-RS dataset, to evaluate the influence of different features extractors and fusion strategies on the classification performance, we do the same experiments discussed above for UC-Merced dataset. The results are shown in Table 3. The classification results in Table 3 once again prove that the parallel feature fusion strategy is better than the serial feature fusion strategy. On the 40% training ratio, VGG-Net-16 is the best feature extractor, while CaffeNet is the best one on the 60% training ratio.

Table 4 shows the comparison of the classification accuracies between our proposed architecture and the other state-of-the-art methods. As we can see from Table 4, TEX-Net-LF and DCA by addition are the most competitive approaches. TEX-Net-LF is the method described in [43], which constructed an architecture where fusing the features obtained from the texture coded mapped image and the standard RGB image. DCA by addition is also a fusion method, which used the first and second output fully connected layers of the network and employed the DCA to fuse the two sets of features [44]. The final experimental results clearly demonstrate that our architecture achieves the highest classification accuracy rate than other state-of-the-art methods.

### 4.5. AID Dataset

On the AID dataset, Table 5 shows the influence of different features extractors and fusion strategies on the classification performance. As we can see from Table 5, the parallel feature fusion strategy is the best fusion method in our architecture. Moreover, using CaffeNet and VGG-Net-16 as feature extractors achieves competitive performance compared to GoogLeNet.

Table 6 shows the classification performance comparison of our architecture compared to the state-of-the-art methods. Our best architecture outperforms all other methods, with an increase in overall accuracy of 1.45% and 1.62% over the second best model using 20% and 50% training ratios, respectively.

### 4.6. NWPU-RESISC45 Dataset

On the NWPU-RESISC45 dataset, Table 7 shows the influence of different features extractors and fusion strategies on the classification performance.  Table 8 shows the classification performance comparison of our architecture compared to the state-of-the-art methods. Our best architecture uses CaffeNet as its feature extractor and employs the parallel feature fusion strategy, which achieves remarkable classification results.

From the classification results on all datasets, we can note that VGG-Net-16 and CaffeNet have the similar performance, while GoogLeNet performs slightly worse. The CaffeNet has only 8 layers, which is much simpler than the VGG-Net-16 and the GoogLeNet with 16 and 22 layers, respectively. From this phenomenon, we can conclude that simpler network performs better. However, we should note that all networks we used are trained on ImageNet whose images are all natural images. Thus, the deeper network (GoogLeNet) is more suitable for processing natural images, which may not be good at processing aerial scenes.

## 5. Conclusion

In this letter, we propose a novel two-stream deep fusion framework for aerial scene classification on high-resolution remote sensing images. In this framework, we firstly use pretrained convolutional neural networks as feature extractor to learn features from the original aerial image and the processed aerial image through saliency detection. Then, the two sets of deep features extracted from the original RGB stream and the saliency stream are fused to one set of features. Finally, the ELM classifier is used for final classification with the fused features. We test our architecture on four challenging datasets. In contrast with other state-of-the-art methods, our proposed architecture can achieve better classification results.

## Figures and Tables

**Table 1:** Classification performance of the proposed method on the UC-Merced dataset using different feature extractors and fusion strategies.

Different architectures	Feature size	Training ratios	
		50%	80%
Without fusion (CaffeNet(RGB))	4096	94.60 ± 0.63	95.69 ± 0.91
Without fusion (CaffeNet(saliency))	4096	92.62 ± 0.74	94.04 ± 0.88
Without fusion (VGG-Net-16(RGB))	4096	94.77 ± 0.73	95.91 ± 1.41
Without fusion (VGG-Net-16(saliency))	4096	92.82 ± 0.91	94.31 ± 0.99
Without fusion (GoogLeNet(RGB))	1024	93.31 ± 0.71	94.99 ± 0.78
Without fusion (GoogLeNet(saliency))	1024	91.32 ± 0.98	93.30 ± 0.55
Fusion strategy 1 (CaffeNet)	8192	95.79 ± 0.52	96.83 ± 0.91
Fusion strategy 2 (CaffeNet)	4096	96.74 ± 0.49	97.80 ± 0.88
Fusion strategy 1 (VGG-Net-16)	8192	96.02 ± 0.77	97.05 ± 1.00
Fusion strategy 2 (VGG-Net-16)	4096	96.97 ± 0.75	98.02 ± 1.03
Fusion strategy 1 (GoogLeNet)	2048	94.46 ± 0.60	96.17 ± 0.90
Fusion strategy 2 (GoogLeNet)	1024	95.41 ± 0.58	97.12 ± 0.96

**Table 2:** Comparison with the state-of-the-art methods on the UC-Merced dataset.

Methods	Training ratios	
	50%	80%
SCK [31]	-	72.52
SPCK [32]	-	73.14
BoVW [33]	-	76.81
BoVW + SCK [31]	-	77.71
BRSP [34]	-	77.80
SIFT + SC [35]	-	81.67 ± 1.23
SSEA [36]	-	82.72 ± 1.18
MCMI [37]	-	88.20
OverFeat [38]	-	90.91 ± 1.19
VLAD [39]	-	92.50
VLAT [39]	-	94.30
MS-CLBP + FV [40]	88.76 ± 0.79	93.00 ± 1.20
CaffeNet [41]	93.98 ± 0.67	95.02 ± 0.81
GoogLeNet [41]	92.70 ± 0.60	94.31 ± 0.89
VGG-VD-16 [41]	94.14 ± 0.69	95.21 ± 1.20
CNN-ELM [27]	-	95.62
salM^3^LBP-CLM [42]	94.21 ± 0.75	95.75 ± 0.80
TEX-Net-LF [43]	95.89 ± 0.37	96.62 ± 0.49
Fusion by addition [44]	-	97.42 ± 1.79
Ours	96.97 ± 0.75	98.02 ± 1.03

**Table 3:** Classification performance of the proposed method on the WHU-RS dataset using different feature extractors and fusion strategies.

Different architectures	Feature size	Training ratios	
		40%	60%
Without fusion (CaffeNet(RGB))	4096	95.79 ± 1.37	96.87 ± 0.66
Without fusion (CaffeNet(saliency))	4096	93.21 ± 1.55	95.86 ± 0.50
Without fusion (VGG-Net-16(RGB))	4096	96.09 ± 0.56	96.64 ± 1.08
Without fusion (VGG-Net-16(saliency))	4096	93.75 ± 0.86	95.55 ± 0.89
Without fusion (GoogLeNet(RGB))	1024	93.77 ± 0.79	95.32 ± 1.92
Without fusion (GoogLeNet(saliency))	1024	91.22 ± 0.78	94.10 ± 1.19
Fusion strategy 1 (CaffeNet)	8192	96.78 ± 1.02	98.00 ± 0.59
Fusion strategy 2 (CaffeNet)	4096	97.74 ± 0.98	98.92 ± 0.52
Fusion strategy 1 (VGG-Net-16)	8192	97.28 ± 0.62	97.81 ± 0.87
Fusion strategy 2 (VGG-Net-16)	4096	98.23 ± 0.56	98.79 ± 0.99
Fusion strategy 1 (GoogLeNet)	2048	94.78 ± 0.77	96.34 ± 1.09
Fusion strategy 2 (GoogLeNet)	1024	95.72 ± 0.87	97.29 ± 1.20

**Table 4:** Comparison with the state-of-the-art methods on the WHU-RS dataset.

Methods	Training ratios	
	40%	60%
Bag of SIFT [45]	-	85.52 ± 1.23
MS-CLBP + BoVW [40]	-	89.29 ± 1.30
GoogLeNet [41]	93.12 ± 0.82	94.71 ± 1.33
VGG-VD-16 [41]	95.44 ± 0.60	96.05 ± 0.91
CaffeNet [41]	95.11 ± 1.20	96.24 ± 0.56
salM^3^LBP-CLM [42]	95.35 ± 0.76	96.38 ± 0.82
TEX-Net-LF [43]	97.61 ± 0.36	98.00 ± 0.52
DCA by addition [44]	-	98.70 ± 0.22
Ours	98.23 ± 0.56	98.92 ± 0.52

**Table 5:** Classification performance of the proposed method on the AID dataset using different feature extractors and fusion strategies.

Different architectures	Feature size	Training ratios	
		20%	50%
Without fusion (CaffeNet(RGB))	4096	87.57 ± 0.32	90.22 ± 0.42
Without fusion (CaffeNet(saliency))	4096	84.45 ± 0.28	87.21 ± 0.48
Without fusion (VGG-Net-16(RGB))	4096	87.24 ± 0.18	90.60 ± 0.31
Without fusion (VGG-Net-16(saliency))	4096	84.25 ± 0.11	87.62 ± 0.56
Without fusion (GoogLeNet(RGB))	1024	84.18 ± 0.53	87.15 ± 0.69
Without fusion (GoogLeNet(saliency))	1024	81.12 ± 0.55	84.28 ± 0.67
Fusion strategy 1 (CaffeNet)	8192	92.26 ± 0.52	94.36 ± 0.29
Fusion strategy 2 (CaffeNet)	4096	92.32 ± 0.41	94.42 ± 0.33
Fusion strategy 1 (VGG-Net-16)	8192	92.04 ± 0.28	94.53 ± 0.18
Fusion strategy 2 (VGG-Net-16)	4096	92.11 ± 0.31	94.58 ± 0.25
Fusion strategy 1 (GoogLeNet)	2048	89.15 ± 0.45	91.25 ± 0.59
Fusion strategy 2 (GoogLeNet)	1024	89.21 ± 0.39	91.31 ± 0.49

**Table 6:** Comparison with the state-of-the-art methods on the AID dataset.

Methods	Training ratios	
	20%	50%
BoVW [42]	-	78.66 ± 0.52
MS-CLBP + FV [42]	-	86.48 ± 0.27
GoogLeNet [41]	83.44 ± 0.40	86.39 ± 0.55
CaffeNet [41]	86.86 ± 0.47	89.53 ± 0.31
VGG-VD-16 [41]	86.59 ± 0.29	89.64 ± 0.36
salM^3^LBP-CLM [42]	86.92 ± 0.35	89.76 ± 0.45
Fusion by addition [44]	-	91.87 ± 0.36
TEX-Net-LF [43]	90.87 ± 0.11	92.96 ± 0.18
Ours	92.32 ± 0.41	94.58 ± 0.25

**Table 7:** Classification performance of the proposed method on the NWPU-RESISC45 dataset using different feature extractors and fusion strategies.

Different architectures	Feature size	Training ratios	
		10%	20%
Without fusion (CaffeNet(RGB))	4096	77.34 ± 0.32	80.54 ± 0.22
Without fusion (CaffeNet(saliency))	4096	75.06 ± 0.51	78.20 ± 0.33
Without fusion (VGG-Net-16(RGB))	4096	77.10 ± 0.14	80.45 ± 0.31
Without fusion (VGG-Net-16(saliency))	4096	74.94 ± 0.23	78.09 ± 0.48
Without fusion (GoogLeNet(RGB))	1024	76.87 ± 0.45	79.12 ± 0.23
Without fusion (GoogLeNet(saliency))	1024	74.67 ± 0.52	77.04 ± 0.19
Fusion strategy 1 (CaffeNet)	8192	80.15 ± 0.23	83.08 ± 0.21
Fusion strategy 2 (CaffeNet)	4096	80.22 ± 0.22	83.16 ± 0.18
Fusion strategy 1 (VGG-Net-16)	8192	79.95 ± 0.12	82.96 ± 0.19
Fusion strategy 2 (VGG-Net-16)	4096	80.03 ± 0.19	83.02 ± 0.14
Fusion strategy 1 (GoogLeNet)	2048	79.69 ± 0.47	81.46 ± 0.22
Fusion strategy 2 (GoogLeNet)	1024	79.75 ± 0.41	81.52 ± 0.28

**Table 8:** Comparison with the state-of-the-art methods on the NWPU-RESISC45 dataset.

Methods	Training ratios	
	10%	20%
GIST [46]	15.90 ± 0.23	17.88 ± 0.22
LBP [46]	19.20 ± 0.41	21.74 ± 0.18
Color histograms [46]	24.84 ± 0.22	27.52 ± 0.14
BoVW + SPM [46]	27.83 ± 0.61	32.96 ± 0.47
LLC [46]	38.81 ± 0.23	40.03 ± 0.34
BoVW [46]	41.72 ± 0.21	44.97 ± 0.28
GoogLeNet [46]	76.19 ± 0.38	78.48 ± 0.26
VGGNet-16 [46]	76.47 ± 0.18	79.79 ± 0.15
AlexNet [46]	76.69 ± 0.21	79.85 ± 0.13
Ours	80.22 ± 0.22	83.16 ± 0.18

## References

[1] Qayyum A., Malik A. S., Saad N. M., Iqbal M., Abdullah M. F., Rasheed W. (2017). Scene classification for aerial images based on CNN using sparse coding technique. *International Journal of Remote Sensing*.

[2] Gan J., Li Q., Zhang Z., Wang J. (2016). Two-level feature representation for aerial scene classification. *IEEE Geoscience and Remote Sensing Letters*.

[3] Yang W., Yin X., Xia G. S. (2015). Learning high-level features for satellite image classification with limited labeled samples. *IEEE Transactions on Geoscience and Remote Sensing*.

[4] Huang F., Yan L. (2015). Hull vector-based incremental learning of hyperspectral remote sensing images. *Journal of Applied Remote Sensing*.

[5] Penatti O. A. B., Nogueira K., Dos Santos J. A. Do deep features generalize from everyday objects to remote sensing and aerial scenes domains?.

[6] Luus F. P. S., Salmon B. P., Van Den Bergh F., Maharaj B. T. J. (2015). Multiview deep learning for land-use classification. *IEEE Geoscience and Remote Sensing Letters*.

[7] Lowe D. G. (2004). Distinctive image features from scale-invariant keypoints. *International Journal of Computer Vision*.

[8] Ojala T., Pietikainen M., Maenpaa T. (2002). Multiresolution gray-scale and rotation invariant texture classification with local binary patterns. *IEEE Transactions on Pattern Analysis and Machine Intelligence*.

[9] Swain M. J., Ballard D. H. (1991). Color indexing. *International Journal of Computer Vision*.

[10] Oliva A., Torralba A. (2001). Modeling the shape of the scene: a holistic representation of the spatial envelope. *International Journal of Computer Vision*.

[11] Sivic J., Zisserman A. Video google: a text retrieval approach to object matching in videos.

[12] Lazebnik S., Schmid C., Ponce J. Beyond bags of features: spatial pyramid matching for recognizing natural scene categories.

[13] Wang J., Yang J., Yu K., Lv F., Huang T., Gong Y. Locality-constrained linear coding for image classification.

[14] Bosch A., Zisserman A., Muñoz X. (2006). Scene classification via pLSA. *Computer Vision—ECCV 2006*.

[15] Blei D. M., Ng A. Y., Jordan M. I. (2003). Latent Dirichlet allocation. *Journal of Machine Learning Research*.

[16] Perronnin F., Sánchez J., Mensink T. (2010). Improving the fisher kernel for large-scale image classification. *Computer Vision–ECCV 2010*.

[17] Jégou H., Perronnin F., Douze M., Sánchez J., Pérez P., Schmid C. (2012). Aggregating local image descriptors into compact codes. *IEEE Transactions on Pattern Analysis and Machine Intelligence*.

[18] Nakada M., Wang H., Terzopoulos D. AcFR: active face recognition using convolutional neural networks.

[19] Ding C., Tao D. (2017). Trunk-branch ensemble convolutional neural networks for video-based face recognition. *IEEE Transactions on Pattern Analysis and Machine Intelligence*.

[20] Batista J. C., Albiero V., Bellon O. R. P., Silva L. AUMPNet: simultaneous Action Units detection and intensity estimation on multipose facial images using a single convolutional neural network.

[21] Jia Y., Shelhamer E., Donahue J. Caffe: convolutional architecture for fast feature embedding.

[22] Szegedy C., Liu W., Jia Y. Going deeper with convolutions.

[23] Wichakam I., Vateekul P. Combining deep convolutional networks and SVMs for mass detection on digital mammograms.

[24] Zhou L., Li Q., Huo G., Zhou Y. (2017). Image classification using biomimetic pattern recognition with convolutional neural networks features. *Computational Intelligence and Neuroscience*.

[25] Huang G. B., Zhu Q. Y., Siew C. K. Extreme learning machine: a new learning scheme of feedforward neural networks.

[26] Tang J., Deng C., Huang G.-B., Zhao B. (2015). Compressed-domain ship detection on spaceborne optical image using deep neural network and extreme learning machine. *IEEE Transactions on Geoscience and Remote Sensing*.

[27] Weng Q., Mao Z., Lin J., Guo W. (2017). Land-Use Classification via Extreme Learning Classifier Based on Deep Convolutional Features. *IEEE Geoscience and Remote Sensing Letters*.

[28] LeCun Y. (1990). Handwritten digit recognition with a back-propagation network. *Advances in neural information processing systems*.

[29] Jingyu G., Yang J., Zhang J., Li M. Natural scene recognition based on convolutional neural networks and deep Boltzmannn machines.

[30] Srivastava N., Hinton G., Krizhevsky A., Sutskever I., Salakhutdinov R. (2014). Dropout: a simple way to prevent neural networks from overfitting. *Journal of Machine Learning Research*.

[31] Yang Y., Newsam S. Bag-of-visual-words and spatial extensions for land-use classification.

[32] Yang Y., Newsam S. Spatial pyramid co-occurrence for image classification.

[33] Castelluccio M. (2015). Land use classification in remote sensing images by convolutional neural networks. *Computer Vision and Pattern Recognition*.

[34] Jiang Y., Yuan J., Yu G. (2012). Randomized spatial partition for scene recognition. *Computer Vision–ECCV 2012*.

[35] Cheriyadat A. M. (2014). Unsupervised feature learning for aerial scene classification. *IEEE Transactions on Geoscience and Remote Sensing*.

[36] Zhang F., Du B., Zhang L. (2015). Saliency-guided unsupervised feature learning for scene classification. *IEEE Transactions on Geoscience and Remote Sensing*.

[37] Ren J., Jiang X., Yuan J. (2015). Learning LBP structure by maximizing the conditional mutual information. *Pattern Recognition*.

[38] Nogueira K., Penatti O. A. B., dos Santos J. A. (2017). Towards better exploiting convolutional neural networks for remote sensing scene classification. *Pattern Recognition*.

[39] Negrel R., Picard D., Gosselin P.-H. Evaluation of second-order visual features for land-use classification.

[40] Huang L., Chen C., Li W., Du Q. (2016). Remote sensing image scene classification using multi-scale completed local binary patterns and fisher vectors. *Remote Sensing*.

[41] Xia G., Hu J., Hu F. (2017). AID: a benchmark data set for performance evaluation of aerial scene classification. *IEEE Transactions on Geoscience and Remote Sensing*.

[42] Bian X., Chen C., Tian L., Du Q. (2017). Fusing local and global features for high-resolution scene classification. *IEEE Journal of Selected Topics in Applied Earth Observations and Remote Sensing*.

[43] Anwer R. M. (2017). Binary patterns encoded convolutional neural networks for texture recognition and remote sensing scene classification. *Computer Vision and Pattern Recognition*.

[44] Chaib S., Liu H., Gu Y., Yao H. (2017). Deep Feature Fusion for VHR Remote Sensing Scene Classification. *IEEE Transactions on Geoscience and Remote Sensing*.

[45] Chen S., Tian Y. (2015). Pyramid of spatial relatons for scene-level land use classification. *IEEE Transactions on Geoscience and Remote Sensing*.

[46] Cheng G., Han J., Lu X. (2017). Remote sensing image scene classification: benchmark and state of the art. *Proceedings of the IEEE*.

[47] Zheng Z., Zhang T., Yan L. (2012). Saliency model for object detection: searching for novel items in the scene. *Optics Expresss*.

[48] Krizhevsky A., Sutskever I., Hinton G. E. (2012). Imagenet classification with deep convolutional neural networks. *Advances in Neural Information Processing Systems*.

[49] Simonyan K., Zisserman A. (2014). Very deep convolutional networks for large-scale image recognition. *Computer Vision and Pattern Recognition*.

[50] Liu C., Wechsler H. (2001). A shape- and texture-based enhanced Fisher classifier for face recognition. *IEEE Transactions on Image Processing*.

[51] Yang J., Yang J.-Y. (2002). Generalized K-L transform based combined feature extraction. *Pattern Recognition*.

[52] Yang J., Yang J.-Y., Zhang D., Lu J.-F. (2003). Feature fusion: Parallel strategy vs. serial strategy. *Pattern Recognition*.

[53] Sheng G., Yang W., Xu T., Sun H. (2012). High-resolution satellite scene classification using a sparse coding based multiple feature combination. *International Journal of Remote Sensing*.

[54] Russakovsky O., Deng J., Su H. (2015). ImageNet large scale visual recognition challenge. *International Journal of Computer Vision*.

